# Complete chloroplast genome sequence and phylogenetic analysis of *Populus maximowiczii* Henry 1913 (Salicaceae Mirb.)

**DOI:** 10.1080/23802359.2024.2392759

**Published:** 2024-08-22

**Authors:** Yan Zhang, Hongyun Shang, Wei Liu, Xiaoliang Shan, Nairui Wang, Rusheng Peng

**Affiliations:** a Laboratory of Poplar Breeding, Liaoning Institute of Poplar Research, Yingkou, China; b Yunnan Key Laboratory for Integrative Conservation of Plant Species with Extremely Small Populations, Kunming Institute of Botany, Chinese Academy of Sciences, Kunming, China; c Department of Plant Pathology, College of Plant Protection, Nanjing Agricultural University, Nanjing, China; d Key Laboratory for Plant Diversity and Biogeography of East Asia, Kunming Institute of Botany, Chinese Academy of Sciences, Kunming, China

**Keywords:** Chloroplast genome, *Populus maximowiczii*, phylogeny

## Abstract

The classification and identification of species in *Populus* has remained a formidable challenge due to widespread interspecies hybridization. The complete chloroplast genome of *Populus maximowiczii* was obtained by Illumina high-throughput sequencing technology, with a typical quadripartite structure and 37.0% GC content. The chloroplast genome of *P. maximowiczii* was 156,892 in length, including a large single-copy region (LSC: 84,988 bp), a small single-copy region (SSC: 16,630 bp), and a pair of inverted repeats (IRs: each 27,637 bp in length). A total of 131 genes were annotated, including 86 protein-coding genes, 37 tRNAs, and 8 rRNAs. The phylogenetic analysis indicated that 43 species belonging to *Populus* were classified into monophyly, with *P. cathayana* being the closest relatives to *P. maximowiczii*. In conclusion, this study provides valuable insights into understanding the phylogeny of *Populus*.

## Introduction

The genus *Populus* L. 1954 has been a taxonomic challenges for a long time due to frequent interspecific hybridization and large intraspecific morphological variation (Wan et al. 2023). *Populus maximowiczii* Henry 1913, a fast-growing *Populus* species commonly used for urban greening (Kaganov 2021), is distributed in China, Russia, Japan, and north Korea. At present, there are few studies on it, which mainly focus on physiology and Interspecific hybridization (Jun et al. 2012; Li and Wang 2019). Chloroplast genome information is of great significance to reveal the origin and evolution of species and their phylogenetic relationships (Gitzendanner et al. 2018). In this study, the chloroplast genome of *P. maximowiczii* was sequenced, assembled, and annotated, and its phylogenetic position in *Populus* was analyzed, which was of great significance for its future genetics and evolutionary studies.

## Materials and methods

**Figure 1.**
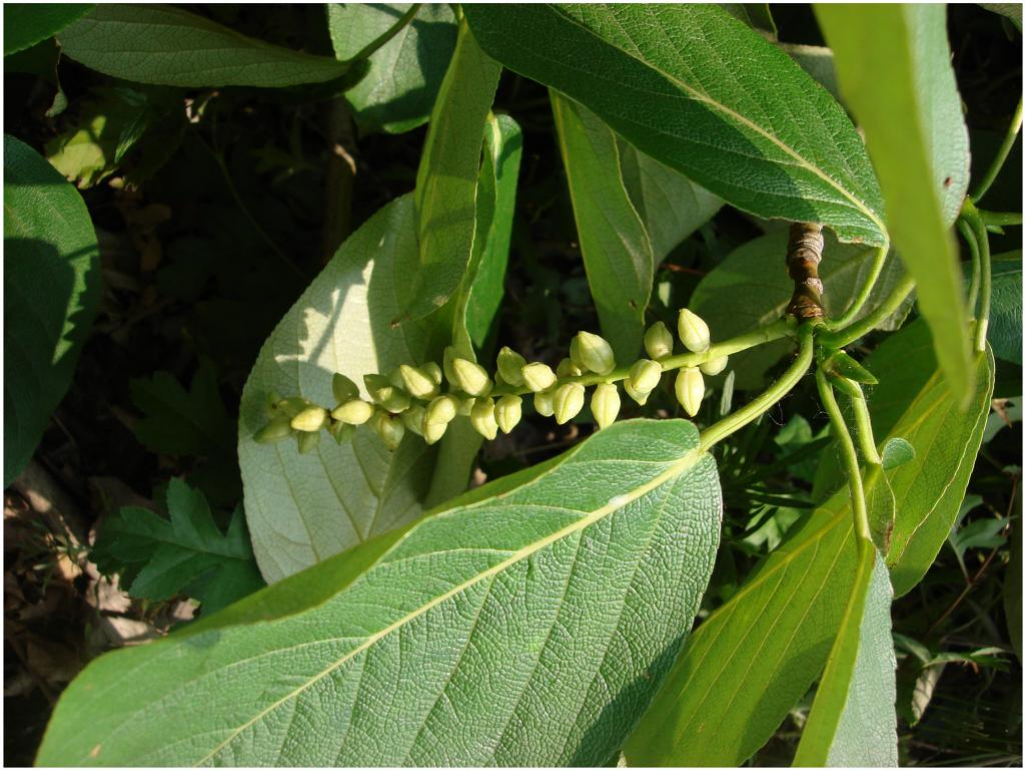
Photo of *Populus maximowiczii*. This photo was taken by the author Yan Zhang in Linghai City, Liaoning province, China. Its buds are conical, brightly colored and viscid. The length of the fruiting catkin is 10–18 cm. Capsule of *Populus maximowiczii* is ovoid-globose and glabrous.

Fresh leaves of *P. maximowiczii* were collected from a individual, which was introduced from Hokkaido, Japan to Linghai City, Liaoning province, China in 2004 (121°22′ E, 41°12′ N) (Figure 1). The voucher specimen was deposited in Molecular Laboratory of Liaoning institute of poplar research, China with specimen number PMM00489 (Yan Zhang, zhangyan913@yeah.net).

**Figure 2.**
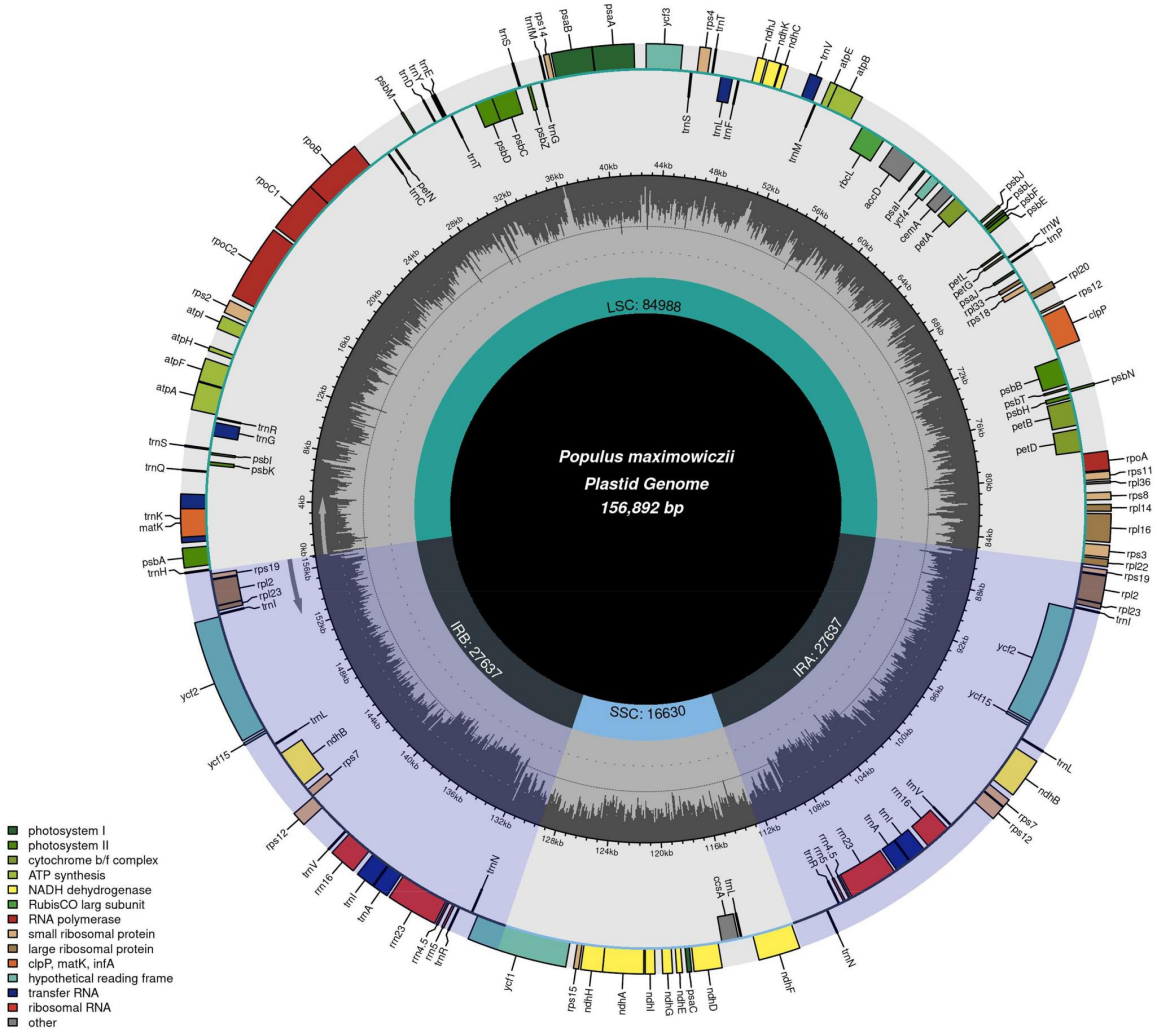
The chloroplast genome map of *Populus maximowiczii* drawn by the online tool Chloroplot. Different functional genes are identified by different colors, with the functions shown at the left bottom. The genes drawn outside of the outer circle are transcribed counter-clockwise, while those drawn inside of the outer circle are clockwise. In the Middle circle, the darker gray represents GC content. In the inner circle, the quadripartite structure (LSC, SSC, IRa, IRb) and the length of each region was drawn.

The genomic DNA for DNA library construction was extracted from about 100 mg fresh leaves using a plant genomic DNA kit (DP305) (Tiangen biotech Inc., Beijing, China) (Xu et al. 2018) in accordance with the instructions. First, the plant tissue was added to 700 μL buffer GP1 and bathed in water at 65 °C for 20 min. Second, 700 μL of chloroform was added and centrifuged at 12,000 rpm for five minutes. Third, the supernatant was added with 700 μL buffer GP2, transferred to spin column CB3 and centrifuged for 30 s. 500 μL buffer GD was added to spin column CB3 and centrifuged for 30 s. Then, 600 μL buffer PW was added and centrifuged for 30 s. The spin column CB3 was dried and 100 μL buffer TE was added to it, and then centrifuged for 2 min after being placed at room temperature for 5 min. Finally, the solution is collected into a collection tube. DNA quality was determined by 1% agarose gel electrophoresis. The chloroplast genome of *P. maximowiczii* was sequenced using the Illumina Hiseq Platform (Genepioneer Biotechnologies, Nanjing, China). Then, it was assembled and annotated using GetOrganelle (Jin et al. 2020) and PGA (Qu et al. 2019), respectively. The online tool Chloroplot (https://irscope.shinyapps.io/ Chloroplot/) was used to draw the chloroplast genome map (Figure 2). CPGview (http://www.1kmpg.cn/cpgview/) was used to generate cis-spliced (Supporting Information Figure S2) and trans-spliced genes (Supporting Information Figure S3) (Liu et al. 2023).

**Figure 3.**
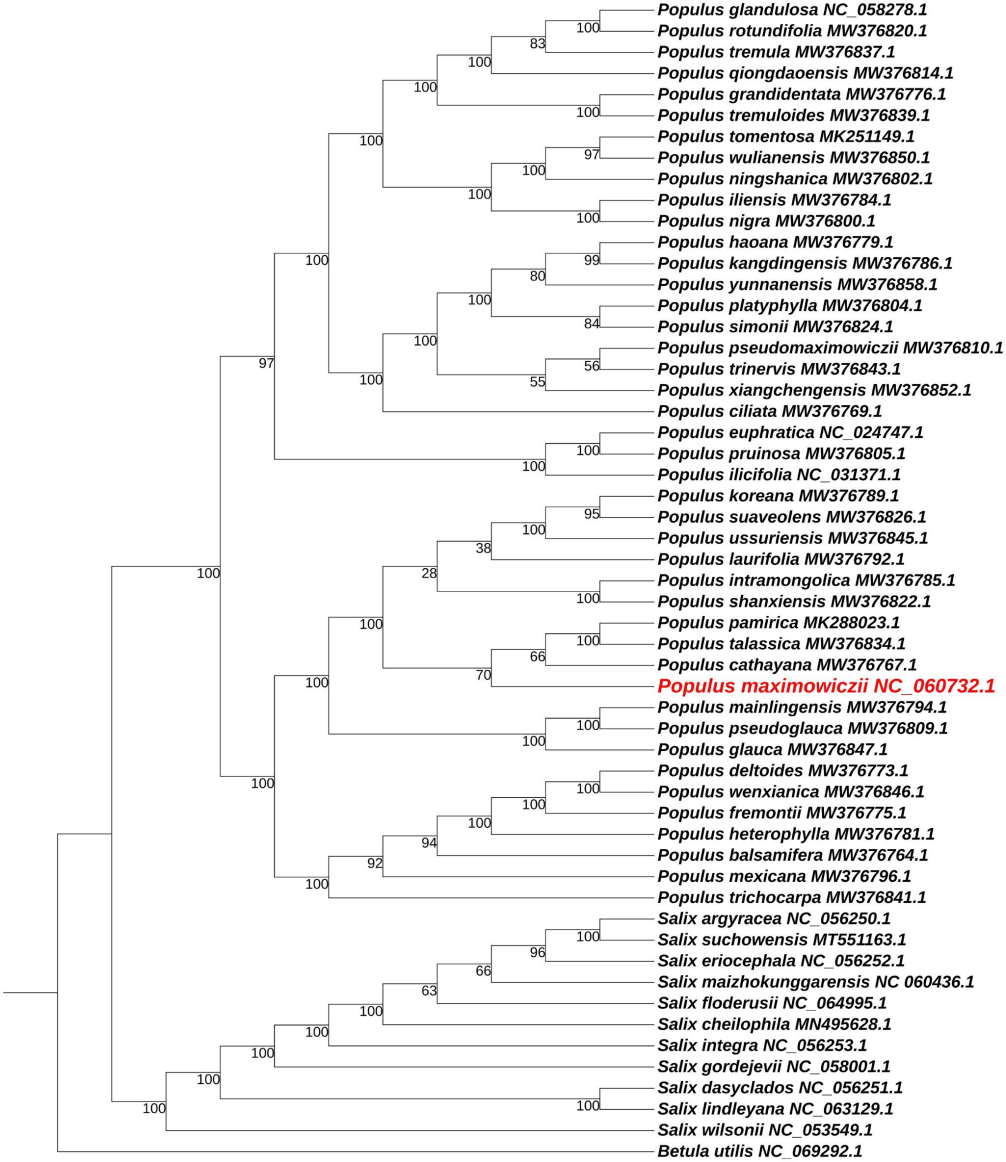
Phylogenetic tree based on the complete chloroplast genome sequences of *Populus maximowiczii* and other 54 species using the maximum likelihood (ML) method, with *Betula utilis* as the outgroup. The numbers below the lines indicate the bootstrap value of each clade. The following sequences download from NCBI were used: *Populus glandulosa* NC_058278.1, *Populus rotundifolia* MW376820.1, *Populus tremula* MW376837.1, *Populus qiongdaoensis* MW376814.1, *Populus grandidentata* MW376776.1, *Populus tremuloides* MW376839.1, *Populus tomentosa* MK251149.1, *Populus wulianensis* MW376850.1, *Populus ningshanica* MW376802.1, *Populus iliensis* MW376784.1, *Populus nigra* MW376800.1, *Populus haoana* MW376779.1, *Populus kangdingensis* MW376786.1, *Populus yunnanensis* MW376858.1, *Populus platyphylla* MW376804.1, *Populus simonli* MW376824.1, *Populus pseudomaximowiczii* MW376810.1, *Populus trinervis* MW376843.1, *Populus xiangchengensis* MW376852.1, *Populus ciliata* MW376769.1, *Populus euphratica* NC 024747.1, *Populus pruinosa* MW376805.1, *Populus ilicifolia* NC 031371.1, *Populus koreana* MW376789.1, *Populus suaveolens* MW376826.1, *Populus ussuriensis* MW376845.1, *Populus laurifolia* MW376792.1, *Populus intramongolica* MW376785.1, *Populus shanxiensis* MW376822.1, *Populus pamirica* MK288023.1, *Populus talassica* MW376834.1, *Populus cathayana* MW376767.1, *Populus mainlingensis* MW376794.1, *Populus pseudoglauca* MW376809.1, *Populus gfauca* MW376847.1, *Populus deltoides* MW376773.1, *Populus wenxianica* MW376846.1, *Populus fremontii* MW376775.1, *Populus heterophylla* MW376781.1, *Populus balsamifera* MW376764.1, *Populus mexicana* MW376796.1, *Populus trichocarpa* MW376841.1, *Salix argyracea* NC_056250.1, *Salix suchowensis* MT551163.1, *Salix eriocephala* NC_056252.1, *Salix maizhokunggarensis* NC_060436.1, *Salix floderusii* NC_064995.1, *Salix cheilophila* MN495628.1, *Salix integra* NC_056253.1, *Salix gordejevii* NC_058001.1, *Salix dasyclados* NC_056251.1, *Salix lindleyana* NC_063129.1, *Salix wilsonii* NC_053549.1, *Betula utilis* NC_069292.1.

To identify the phylogenetic position of *P. maximowiczii*, chloroplast genomes of 54 other species were downloaded from NCBI database, including 42 from *Populus* L., 11 from *Salix* L., and one from Betulaceae Gray. All sequences were aligned using the online service MAFFT (https://mafft.cbrc.jp/alignment/server/) (Katoh et al. 2019). The best nucleotide substitution model was identified using ModelFinder (Kalyaanamoorthy et al. 2017). Based on the TVM + F+R9 model, the maximum-likelihood (ML) tree of *P. maximowiczii* was constructed by the online tool IQTREE (https://www.hiv.lanl.gov/content/sequence/IQTREE/ iqtree.html) (Trifinopoulos et al. 2016) with 1000 bootstrap replicates, and *Betula utilis* D. Don was taken as the outgroup (Figure 3).

## Results

A total of 6.5 Gb clean data was obtained. The reads were then used for de novo assembly of the chloroplast genome and provided an average coverage depth of 4000 x (Supporting Information Figure S1). The overall GC content was 37.0%. The complete chloroplast genome of *P. maximowiczii* had a quadripartite structure with the length of 156,892 bp, consisting of a large single-copy (LSC) region (84,988 bp), a small single-copy (SSC) region (16,630 bp), and a pair of inverted repeats (IRs) (each 27,637 bp in length). A total of 131 genes were annotated, including 86 protein-coding genes, 37 tRNAs, and 8 rRNAs. Among these genes, 10 cis-splicing genes, including *atp*F, *rpo*C1, *ycf*3, *clp*P, *pet*B, *pet*D, *rpl*16, *rpl*2 (two copies), *ndh*B (two copies), and *ndh*A, were discovered, in which *ycf*3 and *clp*P each had two introns, and the other eight genes each had one intron (Supporting Information Figure S2). The trans-splicing gene *rps*12 had three unique exons (Supporting Information Figure S3). The phylogenetic analysis indicated that 43 species belonging to genus *Populus* and 11 species belonging to genus *Salix* were classified as monophyletic groups by 100% bootstrap values, respectively. The genus *Populus* was primarily divided into two clade, with *P. cathayana* Rehd. being the closest relatives to *P. maximowiczii*.

## Discussion and conclusion

The chloroplast genome structure of *P. maximowiczii* was similar to most higher plants (Bock 2007, Yurina et al. 2017). A total of 131 genes of *P. maximowiczii* were identified, which was similar to other *Populus* species, such as *P. trinervis* C. Wang & S. L. Tung (Liu et al. 2020) and *P. rotundifolia* Griff. (Sun et al. 2020). At present, the classification of *Populus* is still in the stage of traditional morphological classification (Wan et al. 2023). Here, the phylogenetic relationships of *P. maximowiczii* and 42 other *Populus* species were constructed based on chloroplast genomes, which provided some useful references for revising the classification of *Populus* at the level of genome. However, the phylogeny of many *Populus* species obtained in this study were inconsistent with morphological taxonomy (Hall and Heybroek 1997). For example, Sect. *Abaso* (*P. mexicana*), Sect. *Leucoides* (*P. heterophylla*), Sect. *Aigeiros* (*P. deltoides, P. fremontii*), Sect. *Tacamahaca* (*P. weinxianica*, *P. balsamifera*, and *P. trichocarpa*) were clustered together. Since chloroplast genomes are maternal inheritance without genetic recombination (Birky 1995; Bock 2007), it is difficult to clarity the relationship between a species and hybrid plants in which this species as female donor. Therefore, the phylogenetic relationship should be constructed by combining nuclear DNA and chloroplast DNA, which may be able to infer the origin of hybrid plants (Liu et al. 2016).

In conclusion, the chloroplast genome of *P. maximowiczii* was successfully sequenced and assembled in this study, which is valuable for assessment and conservation of its genetic resources and provides valuable insights into understanding the phylogeny of *Populus*.

## Supplementary Material

supplemental material-revision (clean).doc

## Data Availability

The chloroplast genome sequence data that support the findings of this study was submitted to GenBank of NCBI (https://www.ncbi.nlm.nih.gov/) with the accession number NC_060732.1. The associated BioProject, Bio-Sample numbers and SRA are PRJNA1058019, SAMN39160095, and SRR27370225, respectively.
