# Supplementary material for: Complete chloroplast genome sequence and phylogenetic analysis of Populus maximowiczii Henry 1913 (Salicaceae Mirb.)
Source: Mitochondrial DNA B Resour. 2024 Aug 22;9(8):1137–41. doi: 10.1080/23802359.2024.2392759 (PMC11342814; doi:10.1080/23802359.2024.2392759)
Supplement: supplemental material-revision (clean).doc [file TMDN_A_2392759_SM1852.doc]

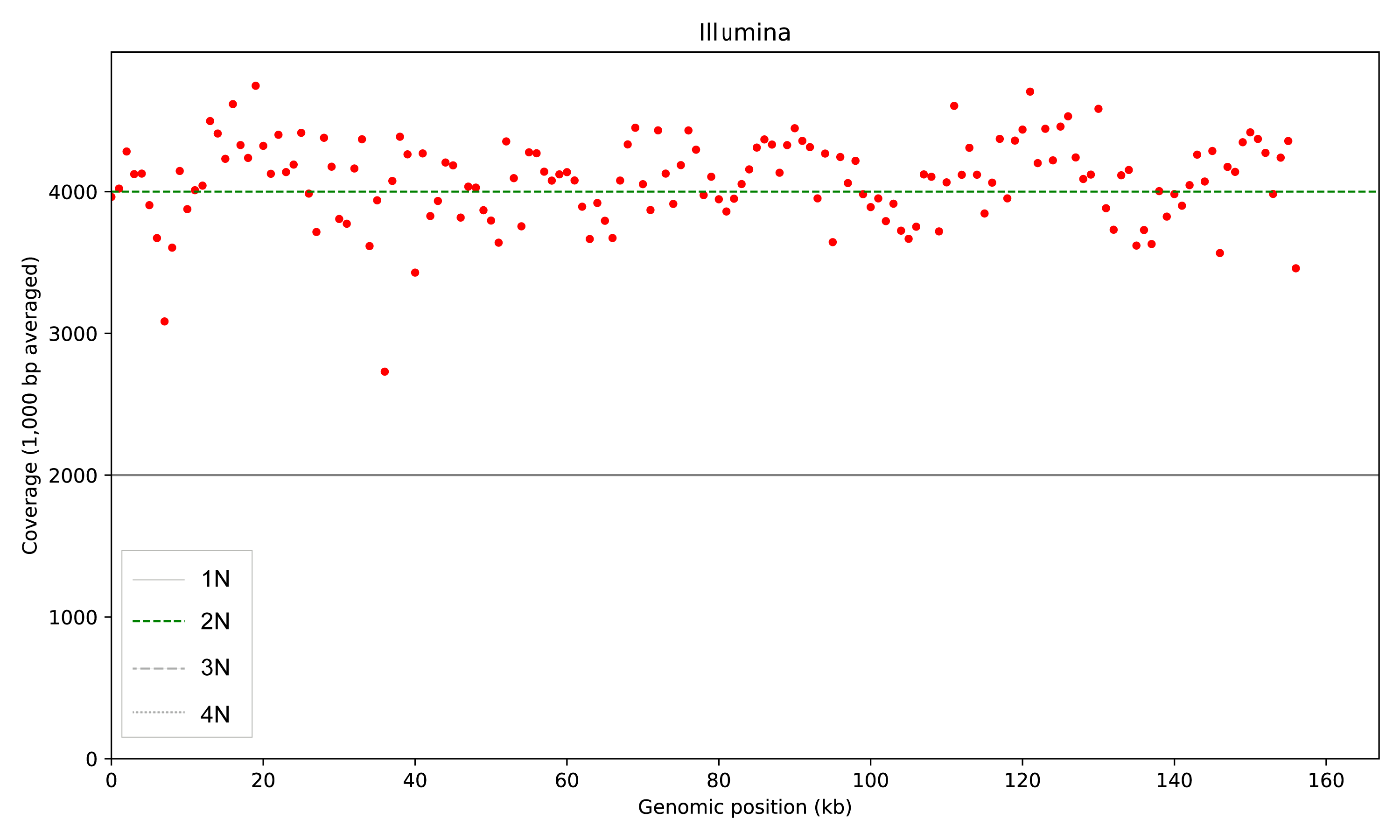


Supplementary Figure 1. The coverage depth of the chloroplast genome of *Populus maximowiczii*.


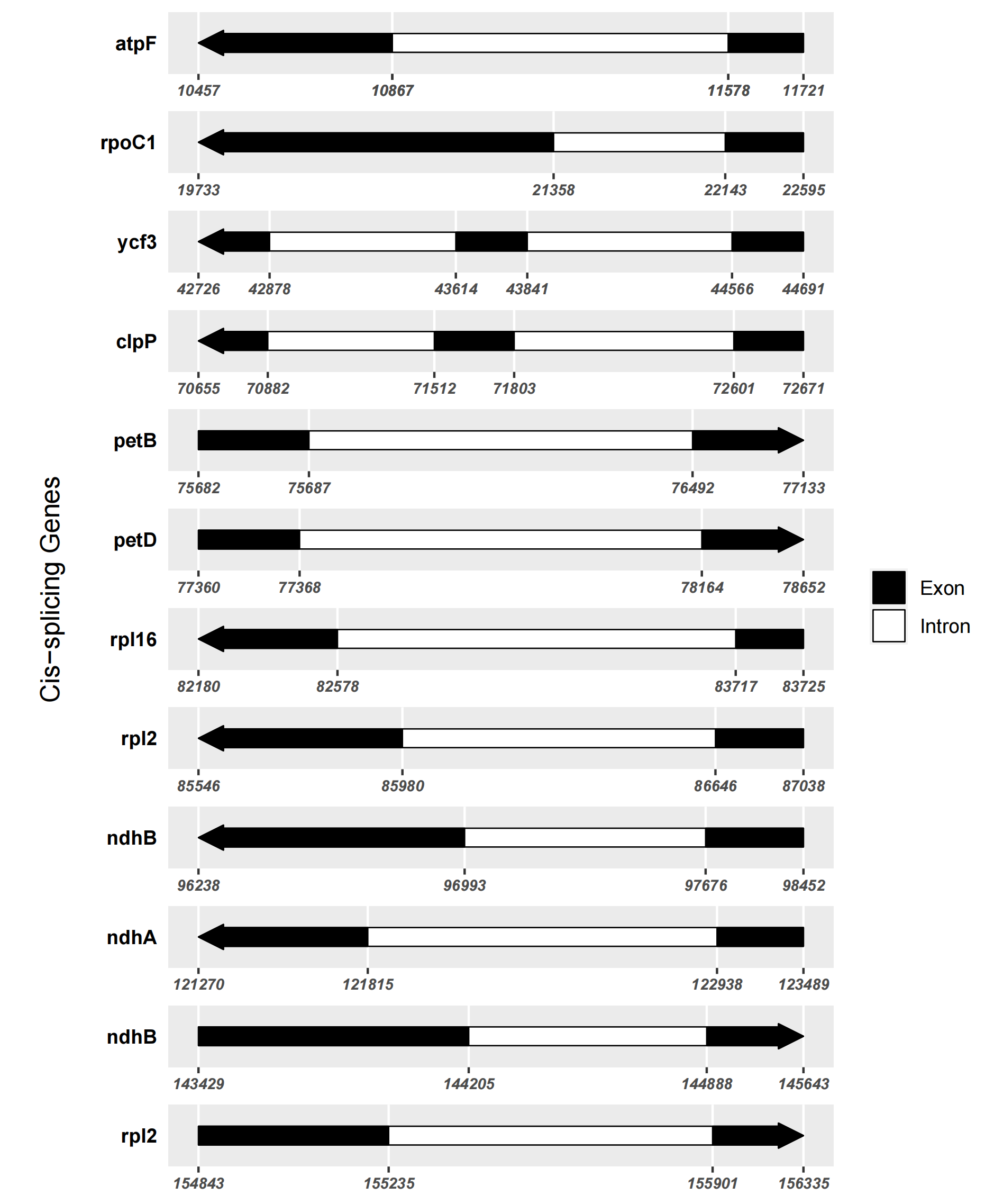


Supplementary Figure 2. Schematic map of the cis-splicing genes in *Populus maximowiczii* chloroplast genome.


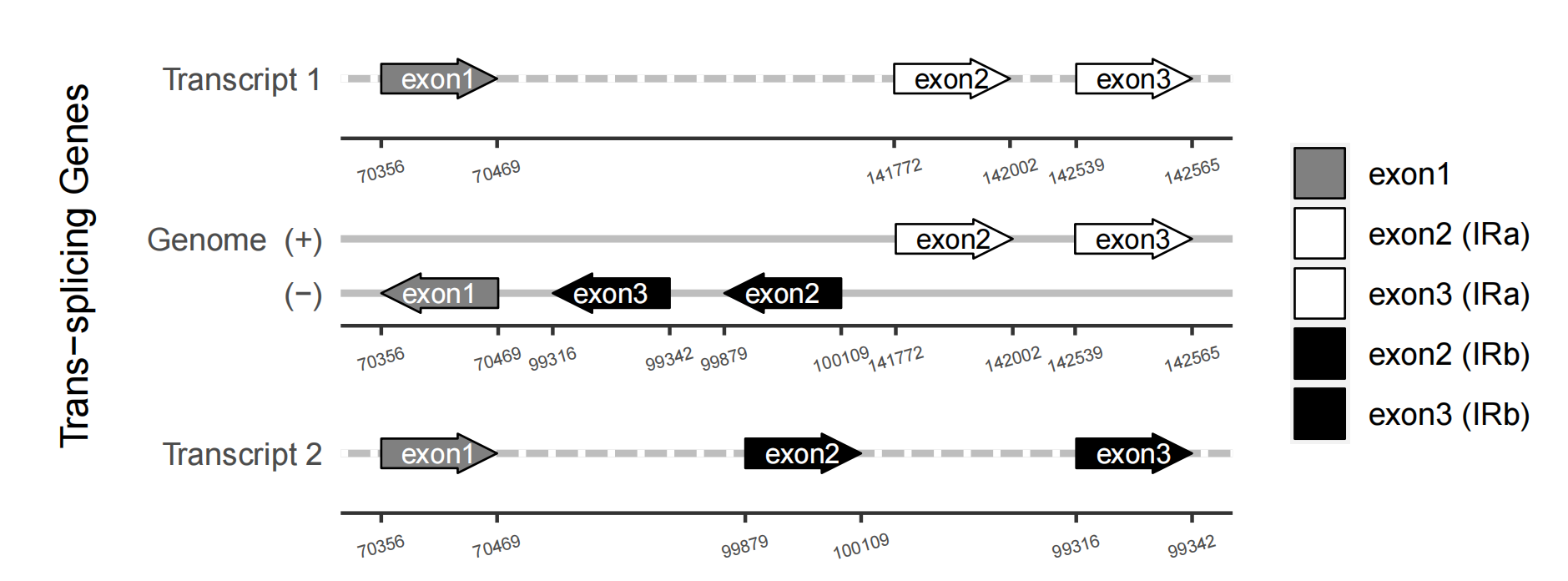


Supplementary Figure 3. Schematic map of the trans-splicing gene in *Populus maximowiczii* chloroplast genome.
